# Early occurrence of childhood dental caries among low literate families

**DOI:** 10.1186/s13104-017-2698-2

**Published:** 2017-08-02

**Authors:** Fatemeh Khani-Varzegani, Leila Erfanparast, Mohammad Asghari-Jafarabadi, Marziyeh Shokravi, Fariba Azabdaftari, Marziyeh Parto, Behjat Shokrvash

**Affiliations:** 1 Department of Health Education and Promotion, School of Health, Tabriz University of Medical Sciences, Golgasht Street, Tabriz, Iran; 2 Department of Pediatric Dentistry, School of Dentistry, Tabriz University of Medical Sciences, Golgasht Street, Tabriz, Iran; 3 Department of Statistics and Epidemiology, School of Health, Tabriz University of Medical Sciences, Tabriz, Iran; 4 Road Traffic Injury Research Center, Tabriz University of Medical Sciences, Tabriz, Iran; 5 Department of Pediatric Dentistry, School of Dentistry, Qazvin University of Medical Science, Qazvin, Iran; 6 Basic Sciences Department, Paramedical School, Tabriz University of Medical Sciences, University Campus, Danshgah Street, Tabriz, Iran

**Keywords:** Oral health, Dental caries, dmft index, Pediatric dentistry, Iran

## Abstract

**Objective:**

The purpose of this cross-sectional study was to identify the oral health status and influencing factors in preschool children in Tabriz, Iran. A total of 756 children from 20% of the total district preschools were selected using a two-step random sampling procedure. Questionnaires were used to collect demographic and socio-economic data. Oral exams were conducted by a single pediatric dentist to assess the children’s oral health, and to determine the number of decayed, missing, and filled teeth (dmft) according to the World Health Organization’s (WHO) definition of caries.

**Results:**

Out of 756 preschool children, 51.5% boys with mean age of years 5.76 (SD = 0.78) were enrolled. The median (25th–75th Pertcentile) of dmft index in boys and girls was 4 (2–9) and 5 (2–8), respectively. Only 15.1% children had decay-free teeth. The results of univariate analysis showed a significant relationship between dmft index and child age (P < 0.001), mother’s years of education (P = 0.001), mother’s employment status (P < 0.001), and family socio-economic status (P < 0.001). On multivariate analysis, statistical significance was found in sex (P = 0.007), age groups except for 5 years (P = 0.210), mother’s education status (P < 0.001) as well as in families with intermediate (P = 0.024), and high (P = 0.072) socio-economic status.

**Electronic supplementary material:**

The online version of this article (doi:10.1186/s13104-017-2698-2) contains supplementary material, which is available to authorized users.

## Introduction

A primary goal of the WHO in 2000 and 2020 was to reduce the prevalence of oral diseases and disabilities and related complications, especially among poor and marginalized populations [1–3].

Oral health Status is determined by using objective and practical, dmft, index provided by the WHO [1]. Fifty percent reduction in decay prevalence among 5–6 year old children, and fewer than 3 dmft index among children under 12 are specific goals of oral health [1, 4].

Research based on dmft index showed that dental decay starts from early childhood in developed [2, 3] and developing countries [5, 6].

Iran, compared with other countries in the East Mediterranean region, has achieved significant progress, except for oral health, in maternal and child health indicators in recent years [7, 8]. Based on existing evidence, Iranians oral health status has been reported poor [9–11], particularly among those in low socio-economic state [10]. Because of limited evidence of oral health among children under 7–12 years, it is necessary to develop context based interventions to achieve optimal oral health among children. This study was designed to identify the oral health status and related factors among preschool and kindergarten children in Tabriz; a large city in Iran. It is expected that the findings of this study will be useful in developing and promoting local policies and effective interventions to achieve WHO oral and general health goals.

## Main text

### Methods

#### Sample and data collection

After obtaining permission from Tabriz University of Medical Sciences Research Ethics Committee (TBZMED.REC.1394.136), logging in preschools was possible through coordination with the five regional education officials.

Based on the previous studies, the estimated sample size according to the previous studies [12, 13], was 384 (α = 0.05 and β = 0.20). Regarding to the design effect, 768 samples would be sufficient. The stratified random sampling method was used for sampling. First, a full list of pre-schools and kindergartens from five districts of education offices was prepared. Then 15 preschools, 20% out of the district preschools, were randomly selected. All 4–7 year old children from 15 preschools and kindergartens were enrolled for the study. Only 12 children were excluded due to unavailability for data collection, child refusal or lack of parental consent (Additional file 1).

#### Material and tools

A questionnaire and a consent form were given to each child to take home for their parents to complete. The demographic questionnaire consisted of characteristics such as age, sex, parental occupation and education years and also family socio-economic status. Family affluence scale [14] was applied to determine the socio-economic status of the family. The scale was earlier validated [15] and consisted of variables such as Iranian family belongings, the child’s personal bedroom, television set(s), refrigerator(s), laundry machine(s), dishwasher(s), computer(s), and fixed telephone(s), etc. Responses were categorized from 0 to 3 and more based on the presence or absence of each belonging. The total score of family belongings was 0 to +14 set in 3 groups: poor (<10), medium (11–13) and good (14 and more).

The evaluation of a Childs’ oral health was performed using the dmft index. After obtaining parental consent, oral examination of each child was performed by a single pediatric dentist at the school. Each child was examined using a probe and a mirror upon brushing his or her teeth. The decayed, missing and filled teeth were counted and the number recorded according to WHO optimal indicator as “50 percent decay free teeth among children” [4].

The type of dental decay was defined according to WHO’s definition of dental caries [16]. In the case of observing damage on the smooth surfaces of the teeth or whitened grooves because of underlying emptiness or softness of the enamel, the tooth was considered as a decayed tooth. In addition, each tooth with temporary filling material or filling and caries again was also considered a decayed tooth.

#### Data analysis

Data were presented using mean (M), standard deviation (SD) and median (25th–75th Percentile) and percentage. To assess the differences between groups, Chi square test and independent sample T test were used for analysis. The Kruskal–Wallis was used to test the differences between the oral health status of children based on dmft index and variables such as child age, mother employment, and family socio-economic status, followed by post hoc tests after adjusting for multiple comparisons by Bonferoni method. The Mann–Whitney U test was used to investigate the differences between dmft index and sex of a child, and maternal education level. A multivariate analysis using quantile regression was used to assess the underlying predictors of dmft (child sex, child age, mother education, mother employment and FAS) (Additional file 2).

Data were analyzed using SPSS15 and STATA11 software and P value of less than 0.05 was considered significant.

### Results

#### Participant characteristics

Out of the 756 preschool children, 51.5% were boys, of mean age 5.76 (SD = 0.78), and the age of mothers was 33 (SD = 5.16). More than half (61.5%) of the mothers had less than 12 years education. Most of the mothers (77.6%) were housewives. Nearly half of the children (44.4%) were in good socio-economic status based on the family property scale (Table 1).

**Table 1:** Sample characteristics

	All (n = 756)	Boy (n = 389)	Girl (n = 367)	P value
Child age (years)	n (%)	n (%)	n (%)	0.975*
4	53 (7.0)	27 (50.1)	26 (49.1)	
5	185 (24.5)	95 (51.4)	90 (48.6)	
6	405 (53.6)	209 (51.6)	196 (48.4)	
7	113 (14.9)	58 (51.3)	55 (48.7)	
M (SD)	5.76 (0.78)	5.76 (0.77)	5.76 (0.79)	
Mother education (years)				0.650**
0–12	465 (61.5)	243 (62.4)	220 (60)	
≥13	291 (38.5)	146 (37.6)	147 (40)	
M (SD)	12.43 (3.56)	12.24 (3.64)	12.63 (3.45)	
Mother employment				0.650**
Housewife	587 (77.6)	307 (78.9)	280 (76.3)	
Employed out of home	149 (19.7)	73 (18.8)	76 (20.7)	
Employed at home	20 (2.6)	9 (2.3)	11 (3)	
FAS				0.002**
Low (≤10)	255 (33.7)	148 (38)	107 (29.2)	
Intermediate (11–13)	336 (44.4)	149 (38.3)	187 (51)	
High (≥14)	165 (21.8)	92 (23.7)	73 (19.9)	
M (SD)	11.39 (2.93)	11.38 (2.95)	11.46 (2.74)	

*M (SD)* mean (standard deviation), *FAS* family affluence scale

* P value based on Independent Sample T test

** P value based on Chi square Test

#### Children oral health status, dmft index and associated factors

The oral health status of preschool children based on median decayed, missing and filled teeth or dmft index was 4 (2–8). The median of the dmft index in boys was 4 (2–9) and in girls 5 (2–8) (Table 2). Univariate analysis showed that there was no significant difference between boys and girls with regard to the dmft index (P = 0.675).

**Table 2:** Distribution and comparison of dmft index by sex and age groups

	dmft index Med (25th–75th)	P value
Child sex		0.675*
Boy (51.5%)	4 (2–9)	
Girl	5 (2–8)	
Child age (years)		<0.001**
4	2 (0–4)	
5	3 (2–5)	
6	5 (3–9)	
7	8 (4–11)	
Mother education (years)		<0.001*
0–12	6 (3–10)	
≥13	3 (1–6)	
M (SD)	12.63 (3.45)	
Mother employment		<0.001**
Housewife	5 (2–9)	
Employed out of home	3 (1–6)	
Employed at home	4.5 (3–9.25)	
FAS		<0.001**
Low (≤10)	6 (3–10)	
Intermediate (11–13)	4 (2–7)	
High (≥14)	4 (2–7)	

*dmft index* decay, missing, filled teeth index, *Med* (*25th–75th*) median (25th–75th Percentile), *M* (*SD*) mean (standard deviation), *FAS* family affluence scale

* P value based on nonparametric Mann–Whitney U Test

** P value based on nonparametric Kruskal–Wallis Test

The relationship between the dmft index and age of children was significant (P < 0.001) (Table 2). The post hoc analysis indicated that the differences among all the age groups was significant (P < 0.001), except for age groups between 4 and 5 years.

Univariate analysis also showed the significant relationship between mother’s education years and child dmft index (P < 0.001). dmft index was lower in children whose mothers’ education years were more than 12. The post hoc analysis showed that there was significant differences between maternal education under 12 years and over 12 years (adjusted P < 0.001).

According to the results of the current study, there is a significant relationship between the dmft index and socio-economic status, based on the “family property index” (P < 0.001). The post hoc analysis revealed that dmft index was significant among all groups (Table 2).

Only 15.1% of the children had decay free teeth (Table 3).

**Table 3:** Distribution of dental decay prevalence among pre-school children by sex and age group

Age (years)	All		Boy		Girl	
	756 (%100)		380 (%51.5)		367 (%48.5)	
	Dental decay free N (%)	Dental decay ≥1 N (%)	Dental decay free N (%)	Dental decay ≥1 N (%)	Dental decay free N (%)	Dental decay ≥1 N (%)
4	14 (26.9)	38 (73.1)	6 (23.1)	20 (76.9)	8 (30.8)	18 (69.2)
5	41 (22.0)	141 (78.0)	25 (26.0)	71 (74.0)	16 (17.8)	74 (82.2)
6	50 (12.3)	355 (87.7)	28 (13.3)	183 (86.7)	22 (11.3)	172 (88.7)
7	10 (18.8)	103 (91.2)	8 (14.3)	48 (85.7)	2 (3.5)	55 (96.5)
Total	115 (15.2)	641 (84.8)	67 (17.2)	322 (82.8)	48 (13.1)	319 (86.9)

Results of multivariate analysis using quantile regression showed significant relationship between sex (P = 0.007), and age groups except for 5 years old children (P = 0.210), mother education (P < 0.001) and family affluence scale (P = 0.072) (P = 0.024) with dmft, No significant relationship was observed between 5 years old children (P = 0.210), mothers employment (P = 0.38) and dmft index (Table 4).

**Table 4:** Multivariate analysis of underlying predictors of dmft index

	Coefficient	95% (CI)	P value
Child sex			
Boy	Referent	–	–
Girl	1.00	(0.27 to 1.73)	0.007
Child age (years)			
4	Referent	–	–
5	1.00	(−0.56 to 2.56)	0.210
6	2.00	(0.53 to 3.47)	0.008
7	4.00	(2.31 to 5.69)	<0.001
Mothers education (years)			
0–12	Referent	–	–
≥13	−2.00	(−2.94 to −1.06)	<0.001
Mother employment			
Housewife	Referent	–	–
Employed out of home	0.00	(−1.11 to 1.11)	1.000
Employed at home	−1.00	(−3.27 to 1.27)	0.387
FAS			
Low (≤10)	Referent	–	–
Intermediate (11–13)	−1.00	(−1.87 to −0.13)	0.024
High (≥14)	−1.00	(−2.09 to 0.09)	0.072

*95%* (*CI*) 95% (confidence interval), *FAS* family affluence scale

P value based on multivariate analysis by quintile regression

## Discussion

The oral health status of preschool children was identified based on dmft index and detected as high sever with specific pattern compared to the WHO criteria [4] in both male and female preschoolers. In this study the dmft index of preschool children in some cases was higher than those of American [17], Turkish [18], Pakestanian [5] and Chinese children [19] but lower than that of Cambodian children [20].

Unfortunately, only 15.2% of preschool children had decay free teeth, that is 17.2% boys and 13.1% girls. These findings are indicative of undesirable situation among Iranian preschoolers compared to the WHO’s optimal indicator as “50 percent decay free teeth among children” [4].

The contribution of demographic characteristics to the dmft index indicates significant effects based on child sex and age groups, mother’s years of education, and socio-economic status based on the family affluence scale.

The results of univariate analysis showed no significant relationship between dmft index and sex, but results of multivariate analysis showed significant relationship (P = 0.007). The findings of this study are consistent with other studies [5, 21], where girls were identified as the most vulnerable groups regarding oral health. It seems that gender difference interferes with the oral health of Iranian preschool children, and thus should influence the development of intervention approaches.

According to the results of this study, older children encountered poorer oral health status. The dmft index, unlike dental caries free value, was significantly higher among older children. This finding is Consistent with Lahore et al’s results, which showed a 33.3% prevalence of caries in 3 years children, 47.6 and 75% in 4 and 5-year-olds [5]. Also, in other developing countries, the dmft index was 1.7, 2.4 and 3.1, among 3, 4 and 5 year old children, respectively [6]. Another study on 14 to 18 years old in Iran showed the same results [22]. According to Pakshier et al. 98.8% of Iranian adults had some degree of decay and teeth loss [23], increased prevalence of dental caries from early ages, which reflects early onset of unhealthy habits and undesirable oral health.

Univariate and multivariate analyses revealed a significant difference between parental characteristics e.g. education years and children dmft index. These findings emphasize on the role of family, especially parents, in establishing early childhood proper health behaviors including oral health. It seems that the highly educated mother may be equipped with proper oral health knowledge and healthy behavior that will in turn affect child’s oral health. Previous studies have confirmed the effect of parental oral health knowledge [19, 22, 24] and practice on children’s oral health [25]. The children of mothers with higher levels of education, normally resulting from higher socio-economic status suffer less from adverse oral health.

The socio-economic status of the family was detected as one of the significant risk factors that affect children’s oral health. The socio-economic status of the family is strongly related with the occupation and income of the parents [25–27]. It seems that high dmft index among children from low level socio-economic families may be the results of low income, and low literacy status of the parents. Children from high socio-economic families showed low value dmft index or good and optimal oral health, consistent with previous studies [22, 25, 28]. These findings revealed the importance of family income and the services that health insurance companies render in developing countries such as Iran. To achieve optimal oral health, the initiation of early family and community based intervention programs along with appropriate public dental health insurance are recommended.

## Conclusions

The results of the study can be generalized to include all pre-school children in Tabriz city because of efficient sampling and using random sampling method. To combat early occurrence of dental caries (dmft index) aggravating with aging among pre-school children, particularly in low literate families in Iran, requires the development of early contextual and family and community-based interventions and surveillance system to improve children’s oral health followed by general health conditions.

To modify undesirable oral health status among Iranian children requires an overwhelming reform in providing dental health services and insurance facilities from birth to the last stage of life especially among low literacy and socio-economic families.

Dental decay is a progressive process, however, as a measuring index; dmft is a sectional dental status estimator. Thus, to fulfill treatment needs dmft index measurement should be repeated within appropriate time intervals starting from dental eruption. It is recommended that other appropriate indexes be used at alongside dmft index to assess the predictors of oral health among groups with high and/or low degree of dental decay and decay-free ones.

## Limitations

- The application of only one index, dmft, to assess pre-school children oral health status.
- The dmft index as a sectional estimator does not apply the progressive nature of dental decay.
- The dmft index as a negative estimator does not show health progress over time.
- The dmft index does not distinguish the difference in the degree of tooth decay, how high and how low.

## Additional files

**Additional file 1.** STROBE checklist form.

**Additional file 2.** Raw data as supplementary information.

## Abbreviations

dmft index: decayed, missing, and filled teeth index
M (SD): mean (standard, deviation)
Med: median
P: *P* value
WHO: World Health Organization
FAS: family affluence scale
