# Supplementary material for: Early occurrence of childhood dental caries among low literate families
Source: BMC Res Notes. 2017 Aug 2;10:366. doi: 10.1186/s13104-017-2698-2 (PMC5541691; doi:10.1186/s13104-017-2698-2)
Supplement: Supplementary file 1 — Additional file 1. STROBE checklist form. [file 13104_2017_2698_MOESM1_ESM.doc]

STROBE Statement—checklist of items that should be included in reports of observational studies

Title: Early Occurrence of Childhood Dental Caries among low Literate Families

|  | | Item No | Recommendation |
| --- | --- | --- | --- |
| **Title and abstract** | | 1 | (*a*) Indicate the study’s design with a commonly used term in the title or the abstract: A cross- sectional study, is Indicated in the abstract. |
|  |  |  | (*b*) Provide in the abstract an informative and balanced summary of what was done and what was found: The study was done on to identify oral health status based on dmft index and related factors among756 preschool children in Tabriz, Iran; 2015, and was found that early occurrence of dental caries happened mostly among in low literate families, and socioeconomic status. |
| Introduction | | | |
| Background/rationale | | 2 | Explain the scientific background and rationale for the investigation being reported: A primary goal of World Health Organization in 2000 and 2020 was to reduce the prevalence of oral diseases and disabilities and related complications, especially among poor and marginalized populations because dental decay is the most common childhood disease in developing countries including Iran. |
| Objectives | | 3 | State specific objectives, including any pre specified hypotheses: This study was designed to identify the oral health status and related factors among preschool and kindergarten children in Tabriz, a large city in Iran.  Specific objectives: 1. identifying sex and age differences, 2. identifying family rate of literacy differences, and 3. socio economic status differences in oral health.  Hypotheses: Oral health is as an important part of general health, the general health of Iranian children has shown improvement past decades, the question was how has been the status of oral health? |
| Methods | | | |
| Study design | | 4 | Present key elements of study design early in the paper:  Oral health, Dental caries, dmft index, Pediatric dentistry, Children, Iran |
| Setting | | 5 | Describe the setting, locations, and relevant dates, including periods of recruitment, exposure, follow-up, and data collection: Settings: preschools and kindergartens, location: regional education officials, relevant dates: September and January 2015. |
| Participants | | 6 | (*a*) *Cohort study*—Give the eligibility criteria, and the sources and methods of selection of participants. Describe methods of follow-up  *Case-control study*—Give the eligibility criteria, and the sources and methods of case ascertainment and control selection. Give the rationale for the choice of cases and controls  *Cross-sectional study*—Give the eligibility criteria: Healthy pre-schoolers, kindergartens, permitted by the parents.  -The sources: five regional educational centres.  -Methods of selection of participants: Stratified random sampling. First, a full list of pre-schools and kindergartens from five districts was prepared. According to the last available records, 586 preschools and kindergartens covered 21939 boys and girls, were active on research time. Then 15 preschools, 20% out of the district preschools, were selected randomly. All 4-7 year old children from 15 preschools and kindergartens were enrolled. |
|  |  |  | (*b*) *Cohort study*—For matched studies, give matching criteria and number of exposed and unexposed  *Case-control study*—For matched studies, give matching criteria and the number of controls per case? |
| Variables | | 7 | Clearly define all outcomes: exposures, Predictors and effect modifiers.  Outcome: Dental caries based on dmft index, Predictors: children age, sex, mother occupation and education years, and family socio-economic status, and effect modifiers: None  Potential confounders: child sex and age, mother employment, mother education years, socio-economic status.  Give diagnostic criteria: dmft index, 50% dental-decay free. |
| Data sources/ measurement | | 8* | For each variable of interest, give sources of data and details of methods of assessment (measurement). Describe comparability of assessment methods if there is more than one group  A demographic questionnaire consisted of children age and sex, and parental occupation and education years and also family socio-economic status were used.  A valid family affluence scale was applied to determine the family socio-economic status. The scale consisted of belongings of the family, the child’s personal bedroom, television set(s), refrigerator(s), laundry machine (s), dishwasher(s), computer(s), fixed telephone(s), and…. . Answers were categorized between0 to 3 and more based on presence or absence of each belonging. The total score of family belongings was 0 to +14 set in 3 groups: poor (less than 10), medium (11-13) and good (14 and more).  Oral health status of children was examined by a pediatric dentistry and the number of the decayed, missed and filled teeth was counted and the type of dental decay was defined according to WHO definition.  Pattern and severity of dental caries of children were categorized based on WHO criteria; %50 dental-caries free. |
| Bias | | 9 | Describe any efforts to address potential sources of bias? None. |
| Study size | | 10 | Explain how the study size was arrived at: The estimated sample size according to the previous studies, α=0.05 and β= 0.20 was 384. Regarding to the design effect, 768 samples would be sufficient. In all 756 pre-schoolers were examined and parents filed out the questionnaires. Only 12 children were excluded due to the absence on data gathering day, child refusal or lack of parental consent. |
| Quantitative variables | | 11 | Explain how quantitative variables were handled in the analyses:  The mean value of children age and years of mother education(Table1),  Median (25th-75th) dmft scores were used to give an estimate of caries (Table2), groupings were chosen based on children sex and age to identify the differences. |
| Statistical methods | | 12 | (*a*) Describe all statistical methods: To assess the differences between groups, Chi-square test and independent sample T test were used for categorical and numeric variables respectively. In this study descriptive and analytical statistic methods were used.  Non-parametric test (Kruskal-Wallis) was used to test the differences between oral health status of children based on dmft and variables such as child age, mother employment, and family socio-economic status. Mann-WhitneyU test were used to investigate the differences between child dmft and child sex, and maternal education level.  Statistical methods used to control for confounding: A multivariate analysis by quantile regression was used to assess the underlying predictors of dmft (child sex, child age, mother education, mother employment and FAS).  Data were analyzed by using SPSS15 and STATA11 Softwares. |
|  |  |  | (*b*) Describe any methods used to examine subgroups and interactions.  Subgroups were recognised based on children sex and age. |
|  |  |  | (*c*) Explain how missing data were addressed: missing data patterns: There was no missing values in the outcome variable: dmft |
|  |  |  | (*d*) *Cohort study*—If applicable, explain how loss to follow-up was addressed  *Case-control study*—If applicable, explain how matching of cases and controls was addressed  *Cross-sectional study*—If applicable, describe analytical methods taking account of sampling strategy |
|  |  |  | (*e*) Describe any sensitivity analyses |
| Continued on next page  Results | | | |
| Participants | 13* | (a) Report numbers of individuals at each stage of study—e.g numbers potentially eligible, examined for eligibility, confirmed eligible, included in the study, completing follow-up, and analysed: 768 pre-schoolers from 15 preschools and kindergartens potentially eligible, 756 children were enrolled. | |
|  |  | (b) Give reasons for non-participation at each stage: Only 12 children were excluded due to the absence on data gathering day, child refusal or lack of parental consent. | |
|  |  | (c) Consider use of a flow diagram  **Target Population**  **Tabriz Pre-schools, Kindergartens:**  **(n=586 preschool,**  **21939 children)**  Distict 1:(n=159, 4867)  Distict 2:(n=72, 3324)  Distict 3:(n=143, 5837)  Distict 4:(n= 146, 4786)  Distict5:(n=66, 3125)  **Eligible Participants (786 children)**  District 1: (n=170)  District 2: (n=116)  District 3(n=205)  District 4:(n=167)  District 5:(n=110)  **Enrolment**  **Analyzed**  **Entered in the analysis (n=756 children)**  Only 12 children were excluded due to the absence on data gathering day, child refusal or lack of parental consent. | |
| Descriptive data | 14* | (a) Give characteristics of study participants (eg demographic, clinical, social) and information on exposures and potential confounders: Out of 756 preschool children, 51.5% were boys, with mean age of 5.76 (SD=0.78). More than half (61.5 %) of mothers had less than 12 years education. Most of mothers (77.6 %) were housewives. Nearly half of the children (44.4%) were in good socio-economic status. | |
|  |  | (b) Indicate number of participants with missing data for each variable of interest: There was no missing data detected. | |
|  |  | (c) *Cohort study*—Summarise follow-up time (eg, average and total amount) | |
| Outcome data | 15* | *Cohort study*—Report numbers of outcome events or summary measures over time | |
|  |  | *Case-control study—*Report numbers in each exposure category, or summary measures of exposure | |
|  |  | *Cross-sectional study—*Report numbers of outcome events or summary measures  Median (25^th^ -75^th^ percentile) decayed, missing and filled teeth or dmft index was 4 (2-8). The Median (25th -75th percentile) of the dmft index in boys was 4(2-9) and in girls 5(2-8) (Table2). On univariate analysis there was no significant difference between boys and girls regarding the dmft index (p=0.675). Only 15.1% children had decay-free teeth.  dmft index was lower in children whose mothers, education years were more than 12. There was a significant relationship between the dmft index and socio-economic status, based on "family property index".  Results of multivariate analysis by quintile regression showed significant relationship between sex (p=0.007), age groups except for 5years children (p=0.210), mother education(p<0.001) and family affluence scale(P=0.072)(P=0.024) with dmft, No sggnificant relationship was observed between 5 years age children(P=0.210), mothers employment(P=0.38) and dmft index. | |
| Main results | 16 | (*a*) Give unadjusted estimates and, if applicable, confounder-adjusted estimates and their precision (eg, 95% confidence interval). Make clear which confounders were adjusted for and why they were included: adjusted for child age and sex, mother education years, mother employment and family socio-economic status. | |
|  |  | (*b*) Report category boundaries when continuous variables were categorized:  Children dmft index was categorized as 0, 1+ value, children age as 4, 5, 6 and 7 years, and mother’s education years as <12 years and 13+ years. | |
|  |  | (*c*) If relevant, consider translating estimates of relative risk into absolute risk for a meaningful time period. None | |
| Other analyses | 17 | Report other analyses done—eg analyses of subgroups and interactions, and sensitivity analyses | |
| Discussion | | | |
| Key results | 18 | Summarise key results with reference to study objectives:  The oral health status of preschool children based on Median (percentile25-percentile75) decayed, missing and filled teeth or dmft index was 4 (2-8). The Median (25th -75th percentile) of the dmft index in boys was 4(2-9) and in girls 5(2-8) (Table2). On univariate analysis there was no significant difference between boys and girls regarding the dmft index (p=0.675).  The relationship between dmft index and age of children was significant (p<0.001). The post hoc analysis indicated that differences between all the age groups was significant (all adjusted p<0.001), except for between 4 and 5 age groups.  The results of univariate analysis also showed the significant relationship between mother education years and child dmft index(p<0.001). dmft index was lower in children whose mothers, education years were more than 12. The post hoc analysis showed that there was significant differences between maternal education under 12 years and over 12 years (p<0.001). The post hoc analysis showed differences in child dmft index between housewife mothers and employed mothers (all adjusted p<0.001). Results of multivariate analysis by quantile regression showed significant relationship between sex(p=0.007), age groups except for 5years children (p=0.210), mother education(p<0.001) and family affluence scale(P=0.072)(P=0.024) with dmft, no significant relationship was observed between 5 years age children(P=0.210), mothers employment(P=0.38) and dmft index. | |
| Limitations | 19 | Limitations of the study: The limitation was application just one index, dmft, to assess children oral health status. To develop a comprehensive program to achieve optimal general and oral health among children, other oral diseases and disorders and dietary and oral health behaviours along with parental health literacy should be taken into account in further researches.  Taking into account sources of potential bias or imprecision:  Discuss both direction and magnitude of any potential bias: The examination of all children oral health was carried out by a single pediatric dentist, so it was free of inter –observer bias and variability. | |
| Interpretation | 20 | Give a cautious overall interpretation of results considering objectives, limitations, multiplicity of analyses, results from similar studies, and other relevant evidence:  Oral health status of preschool children was identified based on dmft index and detected as high sever with specific pattern compared to WHO criteria in both boy and girl pre-schoolers. The results of univariate analysis showed no significant relationship between dmft index and sex, but results of multivariate analysis showed significant relationship (p=0.007). The findings of this study are consistent with other studies [5, 21], where girls were identified as the most vulnerable groups regarding oral health. It seems that gender difference interferes with the oral health of Iranian preschool children, and thus should influence the development of intervention approaches.  In this study dmft index of preschool children in some cases was higher than American, Turkish, Pakestinian and Chinese children but lower than Cambodian’s. only 15.2 percent of preschool children were of decay free teeth, 17.2 boys and 13.1 present girls. These findings are indicative of undesirable situation among Iranian pre-schoolers compared to WHO optimal indicator as “50 percent decay free teeth among children.  Results of this study showed older children encountered poorer oral health status. Increased prevalence of dental caries from early years reflects early presence of unhealthy habits and undesirable oral health. These findings were Consistent with Lahore et al results.  A significant relationship between parental characteristics (e.g. mother employment and education years) and children dmft index was detected.  It seems that parental oral health knowledge may affect children oral health. These findings emphasize on the role of family, especially mother, in establishing early childhood proper health behaviours including oral health.  It seems that high dmft index among children from low level socio-economic families may be the results of low income, and low literacy status of the parents. Children from high socio-economic families showed low value dmft index or good and optimal oral health. These findings revealed the importance of family income and the services that health insurance companies render in developing countries such as Iran. To achieve optimal oral health, the initiation of early family and community based intervention programs along with appropriate public dental health insurance are recommended. .  The limitation was application just one index, dmft, to assess oral health status.  To develop a comprehensive program to achieve optimal general and oral health among children, other oral diseases and disorders and dietary and oral health behaviours along with parental health literacy should be taken into account in further researches.  To modify undesirable oral health status among Iranian children needs an overwhelming reform in rendering dental health services and insurance facilitates from birth to last stage of life especially among low literate and socio-economic families. | |
| Generalisability | 21 | Discuss the generalizability(external validity) of the study results:  Yes they are. The results of the study can be generalizable to the pre-schoolers in Tabriz city because of efficient coverage of the samples and the random sampling method. | |
| Other information | | | |
| Funding | 22 | Give the source of funding and the role of the funders for the present study and, if applicable, for the original study on which the present article is based  This research was funded by Research and Technology Deputy of Tabriz University of Medical Sciences on which the present article is based? | |

*Give information separately for cases and controls in case-control studies and, if applicable, for exposed and unexposed groups in cohort and cross-sectional studies.

**Note:** An Explanation and Elaboration article discusses each checklist item and gives methodological background and published examples of transparent reporting. The STROBE checklist is best used in conjunction with this article (freely available on the Web sites of PLoS Medicine at http://www.plosmedicine.org/, Annals of Internal Medicine at http://www.annals.org/, and Epidemiology at http://www.epidem.com/). Information on the STROBE Initiative is available at www.strobe-statement.org.
